# Supplementary figures and images for: Profiling the bloodstream form and procyclic form Trypanosoma brucei cell cycle using single-cell transcriptomics
Source: eLife. 2023 May 11;12:e86325. doi: 10.7554/eLife.86325 (PMC10212563; doi:10.7554/eLife.86325)

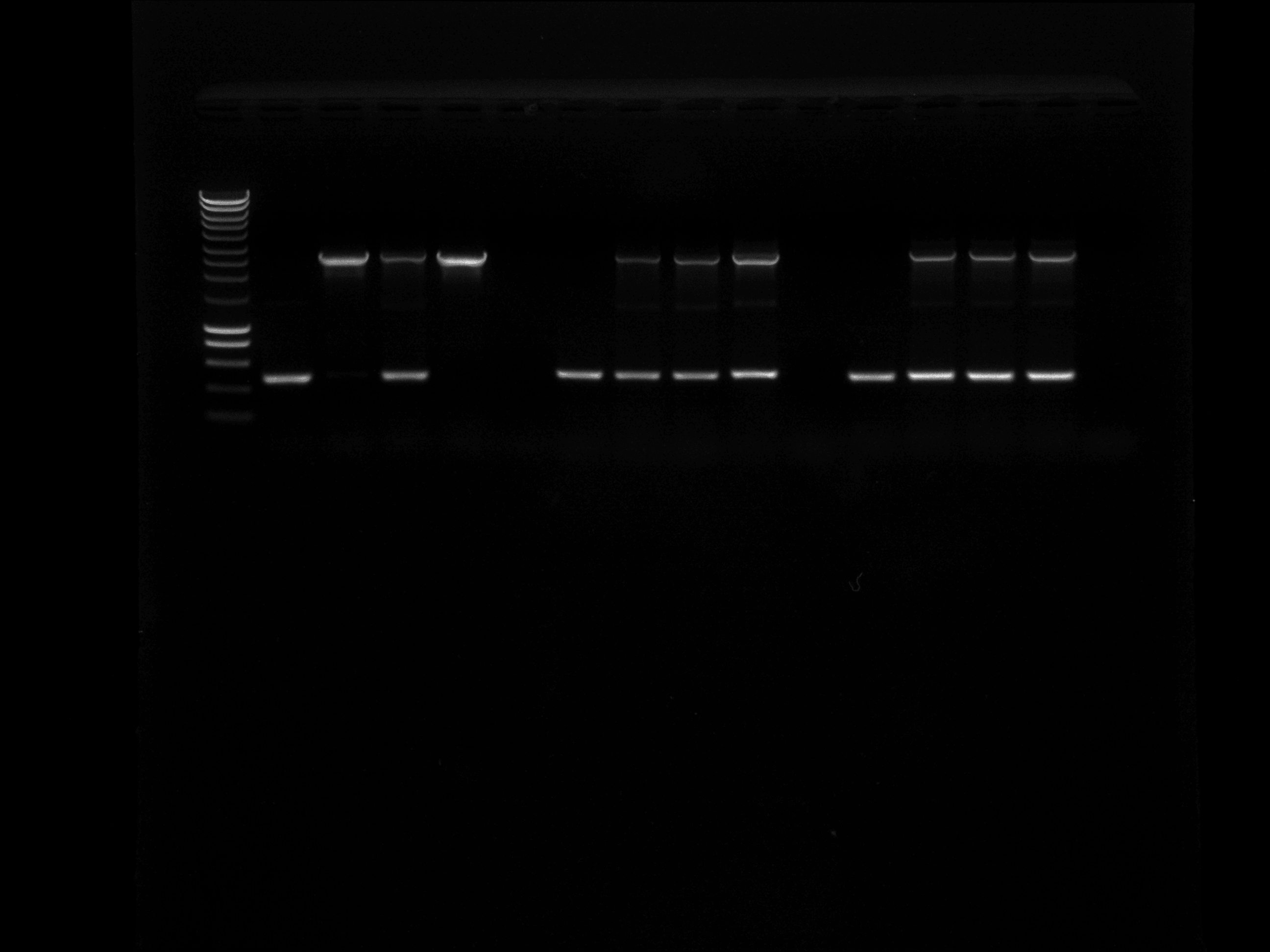

Supplement: Figure 3—figure supplement 1—source data 1. — PCR confirmation of epitope tagged lines. PCR detection of integrated mNG tag fragments for Tb427_060036900, PRI1/Tb427_080028700, and Tb427_110169500. Folder contains uncropped image of gel as PDF as well as original image files. [file elife-86325-fig3-figsupp1-data1.zip › Figure 3ΓÇöfigure supplement 1 - source data 1/CCR_genetag_confir_4_01_23.jpg]
